# In Vivo Wound Healing and Antimicrobial Activities of the Hydroalcoholic Extract and Solvent Fractions of *Arisaema schimperianum* Schott. (Araceae) Tuber in Mice

**DOI:** 10.1155/bmri/6699549

**Published:** 2025-11-09

**Authors:** Betelhem Awoke, Daniel Bisrat, Betelhem Gebreamlak, Abiy Abebe, Sileshi Degu, Kaleab Asres

**Affiliations:** ^1^ Department of Pharmaceutical Chemistry and Pharmacognosy, School of Pharmacy, College of Health Sciences, Addis Ababa University, Addis Ababa, Ethiopia, aau.edu.et; ^2^ Department of Pathology, School of Medicine, College of Health Sciences, Addis Ababa University, Addis Ababa, Ethiopia, aau.edu.et; ^3^ Traditional and Modern Medicine Research and Development Directorate, Armauer Hansen Research Institute, Addis Ababa, Ethiopia, ahri.gov.et

## Abstract

Globally, wounds have become a growing health concern due to the increasing prevalence of vascular diseases. In Ethiopian traditional medicine, plants belonging to the genus *Arisaema* have long been used to treat wounds and infections. This study aimed at evaluating the wound healing and antimicrobial activities of 80% methanol extract and its solvent fractions from the tubers of *Arisaema schimperianum* Schott (Araceae). Wound healing activity was assessed in mice using excision and incision wound models, while skin irritation tests were conducted in rats. The evaluation included measurements of wound contraction rate, epithelialization period, tensile strength, hydroxyproline content, and histological analysis. Antibacterial and antifungal activities were assessed in vitro using the broth dilution method. Ointments formulated at 5% and 10% (*w*/*w*) concentrations were tested for wound healing efficacy. Both concentrations of the methanol extract and its fractions significantly enhanced wound contraction, accelerated epithelialization, and improved tensile strength compared to controls (*p* < 0.001), with the aqueous fraction demonstrating the most potent wound healing effect. These findings were corroborated by histopathological analysis. The aqueous fraction also inhibited the growth of all tested bacterial strains, exhibiting stronger activity against Gram‐positive than Gram‐negative bacteria. The study provides scientific validation for the traditional use of *A. schimperianum* tubers as a natural therapeutic agent for wound management.

## 1. Introduction

Wounds are disruptions in the skin that impair its normal function [1]. The global incidence and healthcare costs of chronic wounds have increased significantly, largely due to the rising prevalence of vascular diseases. Approximately 6 million individuals are estimated to develop chronic wounds each year, placing a financial burden equivalent to 2%–4% of healthcare budgets [2]. In developing regions, chronic wounds affect around 1%–2% of the population over a lifetime [3]. Wounds can result from mechanical injury, chemical exposure, thermal damage, or microbial infections [4]. Although various treatment options exist, many conventional therapies are associated with undesirable side effects [5]. In recent years, herbal remedies have gained attention as potentially safer and more effective alternatives [6].

In Ethiopia, *Arisaema schimperianum* Schott is known by various vernacular names, including *Amoch* in Amharic, *Qolxo* in Sidama, and *Cherana* in Afaan Oromo. It is a herbaceous tuberous plant in the Araceae family and is widely distributed across Ethiopia, the Imatong Hills (on the Uganda–Sudan border), the Democratic Republic of Congo, Kenya, and southern Arabia [7]. It is a seasonal plant that flourishes during the rainy season and remains dormant during the dry months, with its main flowering season occurring in June and July [8].

In traditional Ethiopian medicine, the dried tuber of *A. schimperianum*, often mixed with petroleum jelly, is used to treat wounds associated with glandular tuberculosis, ringworm, scabies, and other skin conditions [9, 10]. In addition to its medicinal uses, the boiled tubers are consumed as food, particularly in southern Ethiopia during periods of drought [11]. The high oxalate content in fresh tubers was identified as a major obstacle to safe consumption. However, Hazo et al. [12] reported that boiling *A. schimperianum* tubers significantly reduces oxalate levels and the associated irritation, thereby allowing occasional consumption.

Despite its widespread traditional use, no prior studies have specifically examined the wound healing and antimicrobial properties of this plant. This study is aimed at filling that gap by investigating the therapeutic potential of *A. schimperianum* tubers. We believe that scientific validation of traditionally used wound healing plants not only advances biomedical research and drug discovery but also reinforces the credibility of traditional medical practices, supporting their integration into modern healthcare.

## 2. Materials and Methods

### 2.1. Plant Material

Fresh tubers of *A. schimperianum* were collected in July 2023 from the Sedie Muja district in the South Gondar Zone, Amhara Region, about 774 km north of Addis Ababa. The plant material was authenticated by the National Herbarium, Department of Plant Biology and Biodiversity Management, College of Natural and Computational Sciences, Addis Ababa University (AAU), where a botanical specimen was deposited (Voucher Number BA‐001) for future reference.

### 2.2. Experimental Animals

Healthy adult Swiss albino mice of either sex (25–30 g, 8–10 weeks old) and female rats (150–250 g) were used for wound healing activity and dermal toxicity studies, respectively. The animals were procured from the Animal House of the School of Pharmacy, AAU, housed under standard laboratory conditions (22°C, 12 h light/dark cycle) with food and water provided ad libitum. All experiments followed internationally accepted guidelines, and animals were acclimatized for 1 week before the study and housed individually [13].

### 2.3. Test Bacteria and Fungi

In vitro antibacterial and antifungal activity tests were performed using clinical or drug‐sensitive standard strains obtained from the Ethiopian Public Health Institute (EPHI) Microbiology Laboratories, Addis Ababa. The Gram‐positive bacteria used were *Staphylococcus aureus* (ATCC 25923), *Staphylococcus epidermidis* (ATCC 12228) and methicillin‐resistant *Staphylococcus aureus* (MRSA) (clinical isolate). *Escherichia coli* (ATCC 25922), *Proteus mirabilis* (ATCC 35659), and *Pseudomonas aeruginosa* (ATCC 27853) were the Gram‐negative bacteria employed. *Candida albicans* (clinical isolate) and *Aspergillus niger* (ATCC 10535) were used as test fungal strains.

### 2.4. Preparation of Hydroalcoholic Extract

Fresh tubers of *A. schimperianum* were cleaned and dried under shade at room temperature. They were then powdered using a mortar and pestle. The powdered tuber (800 g) was soaked in 4 L of 80% methanol for 72 h. The mixture was initially filtered using a nylon cloth, and the marc was remacerated three times (each time for 24 h) to maximize the yield. The combined filtrate was then allowed to pass through Whatman No. 1 filter paper using a pressurized suction system, concentrated under reduced pressure using a rotary evaporator set at 121 mbar, 45 rpm, and a temperature of 40°C. The remaining aqueous solution was dried in an oven at a temperature not exceeding 40°C. The resulting dried extract was weighed, transferred into an amber‐colored vial, and stored in a refrigerator at 4°C until use.

### 2.5. Preparation of Solvent Fractions

Fractionation of the hydroalcoholic extract was performed using solvent–solvent partitioning. The process involved successive solvent–solvent fractionation using *n*‐hexane, chloroform, and water. Initially, the hydroalcoholic extract (30 g) was suspended in 100 mL of distilled water to which an equal volume of *n*‐hexane was added and vigorously shaken in a separatory funnel. After allowing the mixture to stand until clear separation occurred, the process was repeated three times, and the *n*‐hexane layer was collected. This was followed by the addition of an equal volume of chloroform to the aqueous layer, and the lower chloroform layer was carefully collected in a beaker, repeating the process three times to obtain a chloroform fraction. The remaining upper layer was taken as the water fraction. The organic solvent fractions were concentrated in a rotatory evaporator, while the water fraction was dried in an oven at a temperature not exceeding 40°C. The dried fractions were then packed in airtight containers and labeled and stored in a refrigerator until used for further experiments.

### 2.6. Ointment Formulation

A simple ointment was prepared using the hydroalcoholic extract and solvent fractions following the British Pharmacopoeia formula [14]. Thus, three formulations were prepared: control (simple ointment) and medicated ointments with 5% and 10% (*w*/*w*) extract. Ingredients were melted, blended, cooled, and levigated for uniform consistency. The 5% ointment contained 1 g of test substance in 19 g base, while the 10% ointment had 2 g in 18 g base.

### 2.7. Grouping and Dosing of Experimental Animals

Ten groups of mice, each containing six animals, were used for the excision model. One group served as a negative control, eight groups as test groups, and one group as a positive control. Group I received the simple ointment base (negative control). Groups II and III received 5% and 10% (*w*/*w*) hydroalcoholic extract, respectively. Groups IV and V were treated with 5% and 10% (*w*/*w*) *n*‐hexane fraction, Groups VI and VII with 5% and 10% (*w*/*w*) chloroform fraction, and Groups VIII and IX with 5% and 10% (*w*/*w*) water fraction. Group X received 0.2% *w*/*v* nitrofurazone (positive control). For the incision model, animals were assigned similarly, with the addition of Group XI, which served as an untreated negative control.

### 2.8. Toxicity Studies

#### 2.8.1. Acute Oral Toxicity Study

This study followed the Organization for Economic Cooperation and Development (OECD) guideline 425 [15], using five female mice. In the initial phase, one mouse received 2000 mg/kg of 80% methanol *A. schimperianum* tuber extract, dissolved in 5% Tween 80, via oral gavage after an 8‐h fasting period. Observations were made for 30 min and continued over 24 h, with close attention during the first 4 h. Since no mortality was observed, the same dose was administered to four additional mice the following day. All animals were monitored for 14 days for behavioral changes, including alterations in skin, fur, and eye condition, appetite loss, piloerection, lacrimation, tremors, convulsions, and mortality.

#### 2.8.2. Acute Dermal Toxicity Study

A skin irritation test was performed on three groups of Wistar rats, each comprising three males and three females, to evaluate the 80% methanol extract of *A. schimperianum* tubers. Following a 7‐day acclimatization period, the dorsal skin of each rat (approximately 500 mm^2^) was shaved. A simple ointment was applied to the shaved area of the first group, while 5% and 10% extract ointments were applied thinly and uniformly to the shaved areas of the second and third groups, respectively. The sites were covered with gauze and nonocclusive bandages. After 24 h, the bandages were removed, and the sites rinsed with distilled water [16]. Skin reactions were observed at 1, 24, 48, and 72 h using a standardized scoring system (Table 1).

**Table 1:** System for classifying skin reactions.

**Reaction**	**Score**	**Reaction**	**Score**
Erythema		Edema	
No erythema	0	No edema	0
Very slight erythema	1	Very slight edema	1
Well‐defined erythema	2	Well‐defined edema (edge by define raising)	2
Moderate to severe erythema	3	Moderate edema (raising ~1 mm)	3
Severe erythema (beet redness) to scar formation	4	Severe edema (raising more than 1 mm and extended beyond the area of exposure)	4

The score of primary irritation (SPI) was calculated for each rat as follows:

SPI for each rat=∑erythema and edema grade at 244872,,and  hnumber of observation .

The primary irritation index (PII) was determined as the arithmetic mean of the SPI values from all six rats. The degree of irritation was categorized as follows: negligible (0–0.4), slight (0.5–1.9), moderate (2–4.9), or severe (5–8).

PII=∑SPItest−∑SPIcontrolnumber of animals .

### 2.9. Wound Healing Studies

#### 2.9.1. Excision Wound Model

On the day of wounding, animals were anesthetized with ketamine (50 mg/kg) and diazepam (5 mg/kg) administered intraperitoneally (IP) [4]. The dorsothoracic fur was shaved, and a circular area (~314 mm^2^) was marked and excised using sterilized scissors. After recovery, animals were returned to individual cages, with the day of surgery designated as Day 0. From Day 1, wounds were treated daily with topical ointment applications [1, 17].

##### 2.9.1.1. Measurement of Wound Contraction

Wound closure was assessed every other day by tracing the wound area onto transparent paper and transferring it to graph paper for calculation. The percentage of wound contraction was determined using the initial wound size (314 mm^2^) as 100%:

Percent wound contraction=wound area on Day 0−wound area on Day nwound area on Day 0×100,

where *n* is the number of days, that is, 2nd, 4th, 6th, etc., until complete wound healing.

##### 2.9.1.2. Estimation of Hydroxyproline Content

After 10 days of treatment, granulation tissue was collected on Day 11, cleaned, weighed, and dried at 60°C for 12 h. Dried tissue was hydrolyzed in 6N HCl at 110°C for 24 h [18]. Hydrolysates (1 mL) were analyzed alongside standard hydroxyproline solutions, with absorbance measured at 572 nm using a UV‐Vis spectrophotometer (Jenway 6500, England). Hydroxyproline content was calculated using a calibration curve [17].

##### 2.9.1.3. Preparation of Calibration Curve

**Figure 1:**
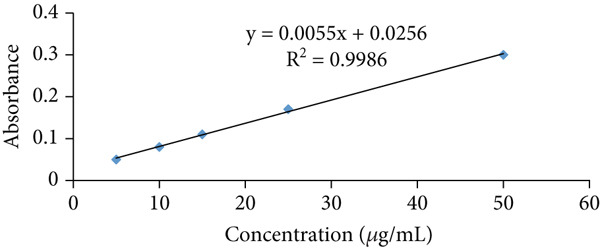
Standard curve for determination of hydroxyproline concentration.

Standard L‐hydroxyproline (0.05 g) was dissolved in 20 mL of HCl and water to prepare a 100 *μ*g/mL stock solution. From this stock, graded concentrations of 5, 10, 15, 25, and 50 *μ*g/mL were prepared for subsequent experiments. Each solution was treated with 1 mL of 0.05 M CuSO₄ and 1 mL of 2.5 N NaOH, incubated at 40°C for 3–5 min, followed by 1 mL of 6% H₂O₂. Then, 4 mL of 3N H₂SO₄ and 2 mL of 5% *p*‐dimethylaminobenzaldehyde were added sequentially, with swirling after each addition. Tubes were capped and heated in a water bath at 70°C for 16 min. After cooling and mixing, absorbance was measured at 572 nm. Unknown hydroxyproline concentrations were determined from the calibration curve (Figure 1) [17].

#### 2.9.2. Incision Wound Model

On the day of incision, animals were anesthetized as previously described. The dorsal fur was shaved, and a 3 cm longitudinal paravertebral incision was made. The wound was sutured using surgical silk (No. 000) and a curved needle (No. 9), with stitches spaced 1 cm apart. From Day 1 postwounding, animals received topical ointment treatment as per the grouping and dosing section (excluding the untreated group). On Day 8, sutures were removed, and treatment continued. Tensile strength was measured on Day 10 using the continuous water flow method, and percent tensile strength was calculated accordingly.

Tensile strength TS of test sample %=TS of test sample−TS of SOTS of SO×100,Tensile strength TS of reference %=TS of referance−TS of SOTS of SO×100,Tensile strength TS of simple ointment SO%=TS of of SO−TS of left untreatedTS of left untreated ×100.

#### 2.9.3. Histopathological Analysis

On Day 12 postwounding, the mice were euthanized using a high dose of IP ketamine and diazepam. Skin samples were collected and fixed in 10% buffered formalin and then processed for paraffin embedding. Sections of 5 *μ*m thickness were prepared and stained with hematoxylin and eosin (H&E) [19]. A light microscope equipped with a digital image analysis system was used to evaluate the stages of wound healing. Parameters such as re‐epithelialization, fibroblast proliferation, collagen deposition, polymorphonuclear cell infiltration, mononuclear cell infiltration, and neovascularization were assessed. Each parameter was graded as mild (+), moderate (++), or marked (+++) to evaluate the extent of epidermal and dermal remodeling [20].

### 2.10. Antimicrobial Activity Test

Mueller–Hinton agar (MHA) and broth (MHB) were used for bacterial cultures, while Sabouraud dextrose agar (SDA) and broth (SDB) were used for fungal cultures. Nutrient broth (NB) served as a general control. Minimum inhibitory concentration (MIC) was determined using the microbroth dilution method in 96‐well plates according to CLSI guidelines [21]. Test samples and controls (ciprofloxacin for bacteria and ketoconazole for fungi) underwent serial dilution. Bacterial cultures were incubated at 37°C for 24 h and fungal cultures at 25°C for 7 days. Growth was assessed using 2,3,5‐triphenyltetrazolium chloride, with MIC defined as the lowest concentration without color change. All tests were conducted in triplicate under sterile conditions [22, 23].

### 2.11. Statistical Analysis

All experimental data are presented as *m* *e* *a* *n* ± *s* *t* *a* *n* *d* *a* *r* *d* *e* *r* *r* *o* *r* *of* *the* *m* *e* *a* *n* (SEM). Statistical comparisons were performed using one‐way analysis of variance (ANOVA), followed by Tukey’s post hoc test. A *p* *v* *a* *l* *u* *e* < 0.05 was considered statistically significant. Data analysis was conducted using SPSS Version 23.

## 3. Results

### 3.1. Yields of Extract and Fractions

The tuber of *A. schimperianum* yielded a 4.75% (*w*/*w*) viscous dark brown hydroalcoholic extract, which was subsequently fractionated into 33.33% *n*‐hexane, 16.66% chloroform, and 50.00% aqueous fractions.

### 3.2. Acute Oral Toxicity Test

A single oral dose of 2000 mg/kg of the hydroalcoholic extract of *A. schimperianum* tuber was found to be safe, with no mortality or observable signs of gross or behavioral toxicity over a 14‐day observation period. The LD₅₀ of the extract is therefore estimated to be greater than 2000 mg/kg.

### 3.3. Acute Dermal Toxicity Test

In the skin irritation study, topical application of a 5% (*w*/*w*) ointment prepared from the 80% methanol extract of *A. schimperianum* tuber did not cause any irritation or visible inflammation. The 10% (*w*/*w*) ointment caused slight redness 24 h post‐application, but no edema or inflammation was noted at 72 h. No signs of toxicity or mortality were observed during the 14‐day monitoring period. Based on the scoring system for classifying skin reactions, their PII values ranged from 0 to 0.4, indicating that the test samples were non‐irritant.

### 3.4. Wound Healing Effects on the Excision Wound

#### 3.4.1. Hydroalcoholic Extract and Solvent Fractions

##### 3.4.1.1. Effects on Wound Contraction

**Figure 2:**
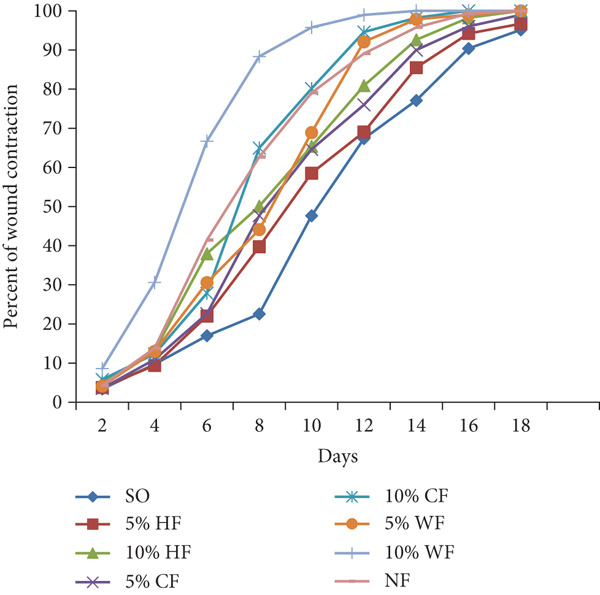
Effect of solvent fractions of *Arisaema schimperianum* tuber on the percentage wound contraction of excision wound model in mice. SO (negative control), simple ointment, HF, *n*‐hexane fraction; CF, chloroform fraction; WF, water fraction; NF (positive control), 0.2% (*w*/*v*) nitrofurazone ointment.

As shown in Table 2, the 10% ointment formulation of the 80% methanol extract and the standard drug induced significant wound contraction (*p* < 0.001 and *p* < 0.05, respectively) from the fourth day post‐wounding compared to the negative control. Among the solvent fractions, the 10% (*w*/*w*) aqueous fraction exhibited a highly significant (*p* < 0.001) wound contraction effect starting from Day 4. All fractions, except the 5% (*w*/*w*) *n*‐hexane fraction, demonstrated significant effects from the sixth day post‐wounding (*p* < 0.01–*p* < 0.001) (Figure 2). The 10% formulations outperformed their 5% counterparts. Complete wound closure was achieved on Day 14 by the 10% aqueous fraction, on Day 16 by the 10% methanol extract, and on Day 18 by the standard drug (Figure 3). In contrast, the simple ointment achieved complete closure only after 20 days.

**Table 2:** Effect of topical application of ointment prepared from the 80% methanol tuber extract of *Arisaema schimperianum* and solvent fractions on wound area (square millimeter) and percent of wound contraction in excision wound model.

**Group**	**Day 2**	**Day 4**	**Day 6**	**Day 8**	**Day 10**	**Day 12**	**Day 14**	**Day 16**	**Day 18**
SO	3.33 ± 2.96 (3.39%)	283.50 ± 2.62 (9.71%)	260.66 ± 2.77 (16.98%)	224.33 ± 4.08 (28.55%)	164.50 ± 7.10 (47.61%)	102.50 ± 4.34 (67.35%)	71.83 ± 4.49 (77.12%)	30.16 ± 2.70 (90.39%)	15.16 ± 2.02 (95.17%)
5% HAE	301.00 ± 4.56 (4.14%)	275.50 ± 2.27 (12.26%)	236.66 ± 2.21 (24.63%)^d2^	192.50 ± 6.61 (38.69%)^a2,d1^	120.00 ± 2.22 (61.78%)^a1^	90.50 ± 8.06 (71.17%)^d2^	33.50 ± 1.76 (89.33)^a1^	12.66 ± 1.22 (95.94)^a1^	5.00 ± 1.06 (98.39)^a1^
10% HAE	297.16 ± 4.57 (5.36%)	262.66 ± 2.29 (16.35%)^a1,b3^	176.33 ± 12.79 (43.83%)^a1,d1,e1^	90.5 ± 5.92 (70.99%)^a1,d1,e1^	50.83 ± 4.36 (83.70%)^a1,d1,e1^	18.33 ± 1.47 (94.12%)^a1,d1,e1^	5.33 ± 0.49 (98.29%)^a1,e1^	0 (100%)^a1,e1^	0 (100)^a1^
5% HF	302.16 ± 4.36 (3.77%)	284.50 ± 2.78 (9.39%)	244.83 ± 2.40 (22.02%)^c1,d1^	189.16 ± 3.15 (39.75%)^a1,c1,d1^	130.16 ± 1.35 (58.54%)	97.16 ± 4.48 (69.05%)^c1,d1^	46.50 ± 3.38 (85.50%)^a1,c3,d1^	18.16 ± 2.02 (94.21%)^a1,c3,d1^	6.16 ± 0.90 (96.71%)^a1,c3,d1^
10% HF	298.33 ± 3.79 (4.99%)	272.66 ± 2.47 (13.16%)	194.83 ± 2.18 (37.95%)^a1,b1^	156.66 ± 2.92 (50.10%)^a1,c1,d1^	108.66 ± 2.89 (65.39%)^a1,b3,c1^	60.00 ± 2.43 (80.89%)^a1,c1,d1^	23.16 ± 11.00 (92.62%)^a1,b1^	5.50 ± 0.42 (98.24%)^a1,b1^	0 (100%)^a1,b1^
5% CF	302.83 ± 2.21 (3.55%)	279.66 ± 1.22 (10.93%)	243.00 ± 2.08 (22.61%)^a2,c1,d1^	170.50 ± 1.38 (47.70%)^a1,b1,c1^	111.00 ± 3.00 (64.64%)^a1,c1^	75.33 ± 5.05 (76.00%)^a1,b1,c1^	30.00 ± 2.81 (90.00%)^a1,b1,c1^	12.33 ± 1.54 (96.04%)^a1,c1,d3^	3.16 ± 0.47 (98.98%)^a1^
10% CF	295.83 ± 3.52 (5.78%)	275.33 ± 1.83 (12.33%)	226.50 ± 2.66 (27.86%)^a1,b2,c1^	110.00 ± 0.68 (64.96%)^a1,b1,d1^	62.33 ± 6.69 (80.14%)^a1,b1,d1^	16.83 ± 0.79 (94.60%)^a1,b1,d1^	5.50 ± 1.20 (98.23%)^a1,b1,d1^	0 (100%)^a1,b^	0 (100%)^a1,b1^
5% WF	301.00 ± 4.56 (4.14%)	273.16 ± 2.97 (13.00%)	218.00 ± 2.22 (30.57%)^a1,b1,c1,d1^	175.50 ± 3.90 (44.10%)^a1,c1,d1^	97 ± 3.47 (68.91%)^a1,a2,c1^	24.67 ± 2.78 (92.09%)^a1,b1,d1^	6.67 ± 0.84 (97.86%)^a1,b1,d1^	0 (100%)^a1,b1^	0 (100%)^a1,b1^
10% WF	285.16 ± 4.57 (8.60%)	216.50 ± 8.92 (30.60%)^a1,b1,c1^	103.83 ± 6.60 (66.72%)^a1,b1,c1,d1^	36.33 ± 1.52 (88.35%)^a1,b1,c1,d1,^	13.16 ± 1.35 (95.78%)^a1,b1,c1,d1^	3.20 ± 0.50 (98.97%)^a1,b1,c1,d1^	0 (100%)^a1,b1,d1^	0 (100%)^a1,b1^	0 (100%)^a1,b1^
NF	300.50 ± 3.30 (4.29%)	270.66 ± 1.76 (13.80%)^a3^	183.83 ± 3.32 (41.45%)^a1,d1^	116.50 ± 2.39 (62.66%)^a1,b1,d1,e1^	65.33 ± 6.60 (79.06%)^a1,b1,d1,e1^	25.00 ± 1.91 (91.98%)^a1,b1,d1,e1^	13.16 ± 1.42 (95.78%)^a1,b1^	2.16 ± 0.47 (98.80%)^a1,b1^	0 (100%)^a1,b1^

*Note:* Data are presented as mean ± SEM (*n* = 6) and analyzed using a one‐way ANOVA with a Tukey post hoc test.

Abbreviations: CF, chloroform fraction; HAE, 80% methanol extract; HF, *n*‐hexane fraction; NF (positive control), 0.2% (*w*/*v*) nitrofurazone ointment; SO (negative control), simple ointment; WF, water fraction.

^a^Compared with SO.

^b^Compared with 5% (*w*/*w*) HF ointment.

^c^Compared with 10% (*w*/*w*) HF ointment.

^d^Compared with NF.

^e^Compared with 5% (*w*/*w*) HAE ointment.

^1^ *p* < 0.001. ^2^ *p* < 0.01. ^3^ *p* < 0.05.

Figure 3Photograph of skin after topical application of (a) 10% (*w*/*w*) ointment prepared from 80% methanol extract of the tuber of *Arisaema schimperianum*, (b) 10% (*w*/*w*) ointment prepared from the water fraction of the tuber of *Arisaema schimperianum*, and (c) 0.2% (*w*/*v*) nitrofurazone ointment for wound contraction test.

**Figure:**
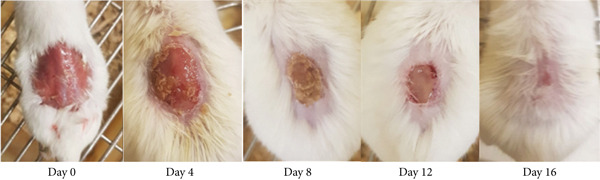
(a)

**Figure:**
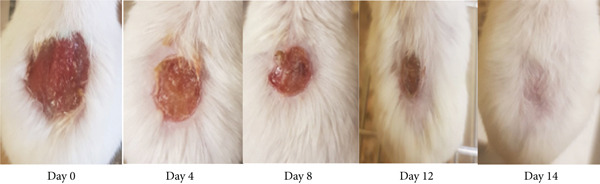
(b)

**Figure:**
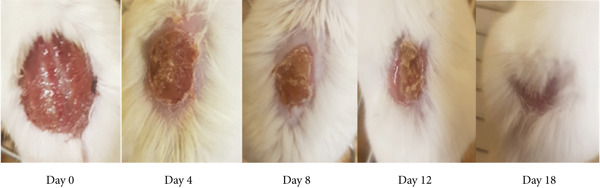
(c)

##### 3.4.1.2. Effect on Epithelialization Period

Table 3 shows that the time required for complete epithelialization was significantly shorter in the extract‐ and standard drug–treated groups compared to the control. Treatment with 10% and 5% (*w*/*w*) aqueous fractions and 10% chloroform fraction significantly reduced the epithelialization period (*p* < 0.01). Similarly, 5% (*w*/*w*) chloroform and 10% (*w*/*w*) *n*‐hexane fractions also showed a significant reduction (*p* < 0.05). Although differences existed between the 5% and 10% preparations, significance was only observed in the water fraction group (*p* < 0.05).

**Table 3:** Effect of topical application of the 80% methanol extract and solvent fractions of *Arisaema schimperianum* tuber on the period of epithelialization.

**Group**	**Epithelialization period (days)**
SO	18.67 ± 0.51
5% HAE	16.00 ± 0.44^a2^
10% HAE	13.00 ± 0.67^a1^
5% HF	17.33 ± 0.42
10% HF	16.00 ± 0.73^a1^
5% CF	16.00 ± 0.51^c1^
10% CF	15.83 ± 0.54^a1^
5% WF	15.67 ± 0.61^b1^
10% WF	13.67 ± 0.61^a1,b1,c2^
NF	14 ± 0.73^a1,b1,c2^

*Note:* Data are presented as mean ± SEM (*n* = 6) and analyzed using a one‐way ANOVA with a Tukey post hoc test.

Abbreviations: CF, chloroform fraction; HAE, 80% methanol extract; HF, *n*‐hexane fraction; NF (positive control), 0.2% (*w*/*v*) nitrofurazone ointment; SO (negative control), simple ointment; WF, water fraction.

^a^Compared with SO.

^b^Compared with 5% (*w*/*w*) HF ointment.

^c^Compared with 10% (*w*/*w*) HF ointment.

^1^ *p* < 0.001. ^2^ *p* < 0.05.

##### 3.4.1.3. Histological Analysis

As detailed in Table 4, histological examination revealed enhanced healing in animals treated with 10% (*w*/*w*) aqueous and chloroform fractions, as well as 10% hydroalcoholic extract (Figures 4a, 4b, and 4c), which exhibited increased fibroblast activity, moderate collagen deposition, neovascularization, and reduced inflammatory cell infiltration compared to controls. Standard drug–treated tissues (Figure 4d) showed high collagen content, moderate fibroblast presence, and improved vascularization with fewer inflammatory cells. Treatments with 5% (*w*/*w*) hydroalcoholic extract and aqueous fraction also demonstrated moderate levels of healing markers (Figure 4e,f), while tissues from the simple ointment group displayed limited fibroblast and collagen presence, increased inflammation, and reduced vascularization (Figure 4j).

**Table 4:** Histological determination of the wound healing process of the 80% methanol extract and solvent fractions of *Arisaema schimperianum* tuber.

**Group**	**RE**	**FB**	**CD**	**PMN**	**MNC**	**NV**
SO	+	+	+	++	++	+
5% HAE	++	++	++	++	++	++
10% HAE	+++	+++	+++	++	+	++
5% HF	++	++	++	++	+++	+
10% HF	++	++	++	+++	++	+++
5% CF	++	++	++	+++	+++	+
10% CF	+++	+++	++	+++	+++	++
5% WF	++	++	++	++	++	++
10% WF	+++	+++	++	+++	+++	++
NF	++	++	+++	++	++	++

*Note:* + (mild), ++ (moderate), and +++ (severe) for epidermal or dermal remodeling.

Abbreviations: CD, collagen deposition; CF, chloroform fraction; FB, fibroblast; HAE, 80% methanol extract; HF, *n*‐hexane fraction; MNC, mononuclear cell; NF (positive control), nitrofurazone 0.2% (*w*/*v*) ointment; NV, neovascularization; PMN, polymorphonuclear cell; RE, re‐epithelialization; SO (negative control), simple ointment; WF, water fraction.

Figure 4Photomicrograph of histopathological section of wound tissue (stained with H&E, 100× magnifications) obtained from mice treated with (a) 10% (*w*/*w*) water fraction of *Arisaema schimperianum* tuber, (b) 10% (*w*/*w*) chloroform fraction of *Arisaema schimperianum* tuber, (c) 10% (*w*/*w*) 80% methanol extract of *Arisaema schimperianum* tuber, (d) nitrofurazone 0.2% (*w*/*v*) ointment, (e) 5% (*w*/*w*) water fraction of *Arisaema schimperianum* tuber, (f) 5% (*w*/*w*) 80% methanol extract of *Arisaema schimperianum* tuber, (g) 10% (*w*/*w*) *n*‐hexane fraction of *Arisaema schimperianum* tuber, (h) 5% (*w*/*w*) chloroform fraction of *Arisaema schimperianum* tuber, (i) 5% (*w*/*w*) *n*‐hexane fraction of *Arisaema schimperianum* tuber, and (j) simple ointment. C, collagen fiber; F, fibroblast; M, macrophage.

**Figure:**
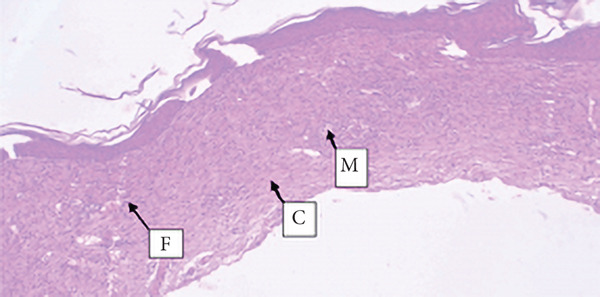
(a)

**Figure:**
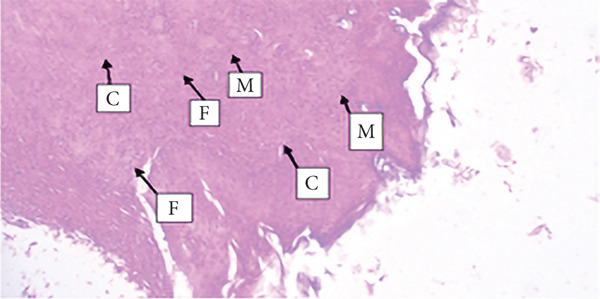
(b)

**Figure:**
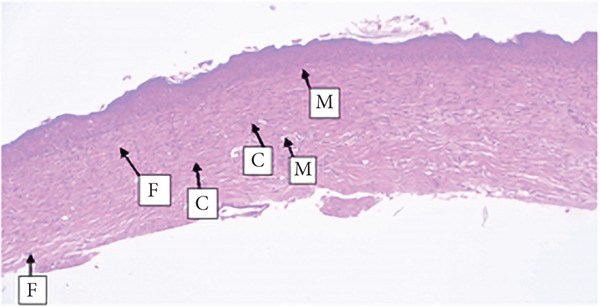
(c)

**Figure:**
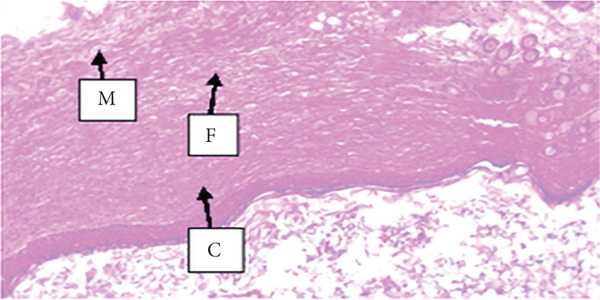
(d)

**Figure:**
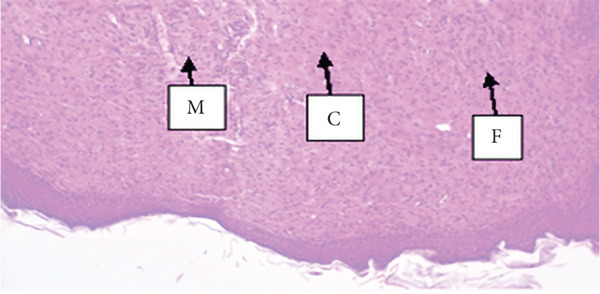
(e)

**Figure:**
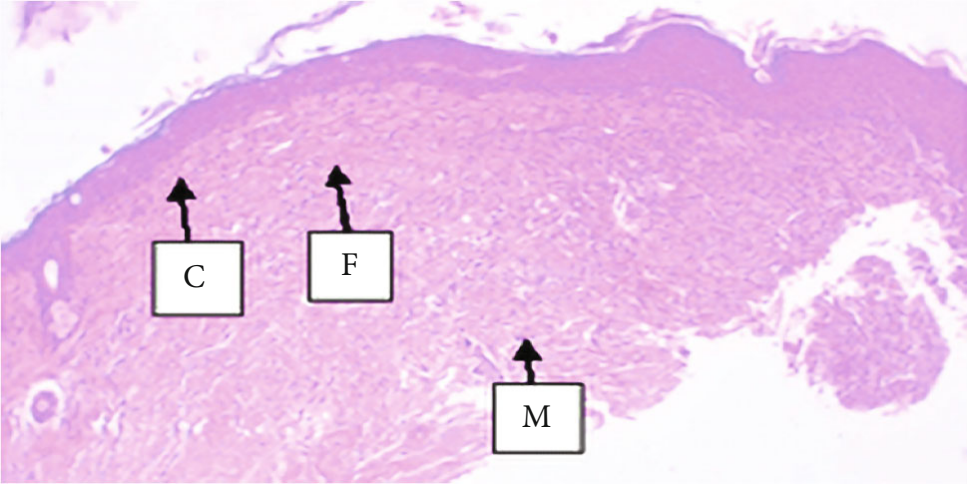
(f)

**Figure:**
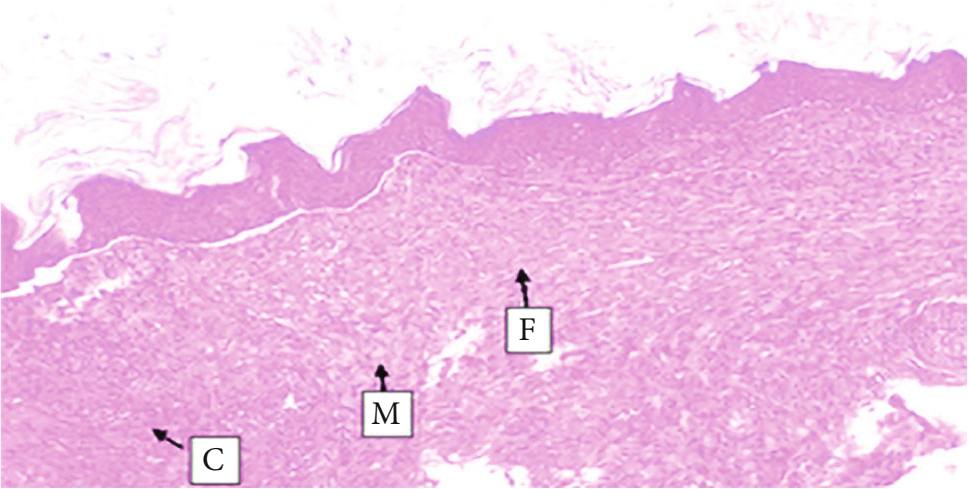
(g)

**Figure:**
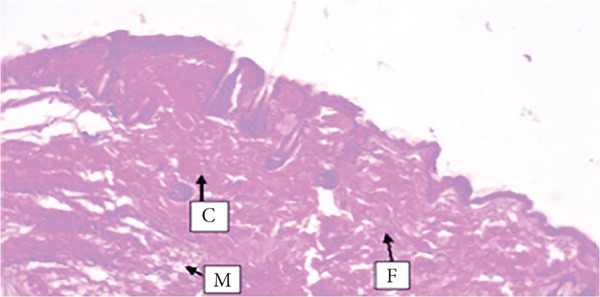
(h)

**Figure:**
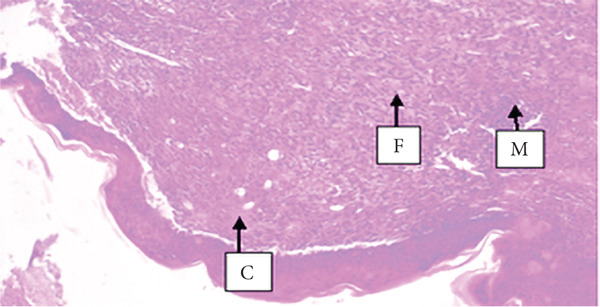
(i)

**Figure:**
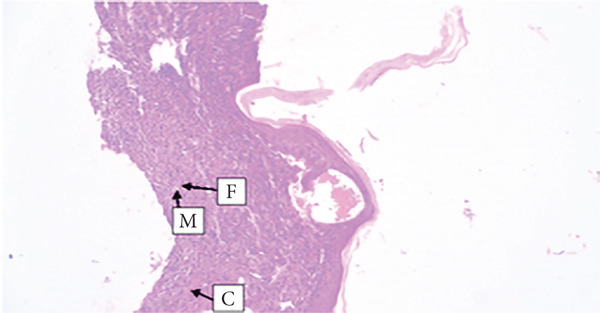
(j)

##### 3.4.1.4. Effects on Tissue Hydroxyproline Content

As shown in Table 5, hydroxyproline levels in granulation tissue were significantly higher (*p* < 0.01) in animals treated with the 10% hydroalcoholic extract and all solvent fractions than in the negative control group. The highest hydroxyproline concentrations were recorded for the 10% aqueous fraction (61.00 mg/g tissue) and the standard drug nitrofurazone (58.02 mg/g tissue). The 10% hydroalcoholic extract and the standard drug showed comparable results.

**Table 5:** Effect of topical application of 80% methanol extract and solvent fractions of *Arisaema schimperianum* tuber on tissue hydroxyproline content in excision wound model.

**Group**	**Hydroxyproline content (mg/g tissues)**
SO	10.00 ± 0.57
5% HAE	41.00 ± 2.23^a1,d1^
10% HAE	58.67 ± 1.15^a1,e1^
5% HF	12.33 ± 1.20^c1,d1^
10% HF	31.33 ± 1.29^a1,b1,d1^
5% CF	28.00 ± 1.57^a1,b1,d1^
10% CF	34.33 ± 0.881^a1,b1,d1^
5% WF	43.67 ± 1.20^a1,b1,c1,d1^
10% WF	61.00 ± 2.30^a1,b1,c1,d1^
NF	58.02 ± 1.11^a1,b1,c1,e1^

*Note:* Data are presented as mean ± SEM (*n* = 6) and analyzed using a one‐way ANOVA with a Tukey post hoc test.

Abbreviations: CF, chloroform fraction; HAE, 80% methanol extract; HF, *n*‐hexane fraction; NF (positive control), 0.2% (*w*/*v*) nitrofurazone ointment; SO (negative control), simple ointment; WF, water fraction.

^a^Compared with SO.

^b^Compared with 5% (*w*/*w*) HF ointment.

^c^Compared with 10% (*w*/*w*) HF ointment.

^d^Compared with NF.

^e^Compared with 5% (*w*/*w*) HAE ointment.

^1^ *p* < 0.001.

### 3.5. Wound Healing Effects on Incision Wound

#### 3.5.1. Effects on Tensile Strength

As shown in Table 6, the 5% and 10% ointments of the 80% methanol extract and the standard drug significantly increased (*p* < 0.001) the tensile strength of incision wounds compared to the negative control and untreated groups. All solvent fractions, except for the 5% *n*‐hexane fraction, also significantly increased tensile strength (*p* < 0.001) relative to the simple ointment. The 5% *n*‐hexane fraction‐treated group showed a moderate but significant increase (*p* < 0.05). No significant difference was observed between the 10% hydroalcoholic extract and the standard drug.

**Table 6:** Effect of topical application of the 80% methanol extract and solvent fractions of *Arisaema schimperianum* tuber on tensile strength.

**Groups**	**Tensile strength (g)**	**Percent tensile strength (%)**
Untreated control	187.50 ± 7.04	—
SO	230.83 ± 19.07	18.86
5% HAE	304.50 ± 15.01^a1,d2,e1^	31.91
10% HAE	425.00 ± 18.02^a1,d1,e1^	84.11
5% HF	276.66 ± 18.95^c2,d1^	19.85
10% HF	333.33 ± 17.44^a2,b1^	44.40
5% CF	340.00 ± 6.83^a2,b1^	47.29
10% CF	378.33 ± 18.87^a1,b1^	63.89
5% WF	359.16 ± 9.69^a1,b1^	55.59
10% WF	440.00 ± 32.04^a1,b1,c1^	90.61
NF	406.67 ± 16.67^a1,b1,c1,e1^	76.32

*Note:* Data are presented as mean ± SEM (*n* = 6) and analyzed using a one‐way ANOVA with a Tukey post hoc test.

Abbreviations: CF, chloroform fraction; HAE, 80% methanol extract; HF, *n*‐hexane fraction; NF (positive control), 0.2% (*w*/*v*) nitrofurazone ointment; SO (negative control), simple ointment; WF, water fraction.

^a^Compared with SO.

^b^Compared with untreated control.

^c^Compared with 5% (*w*/*w*) HF ointment.

^d^Compared with NF.

^e^Compared with 5% (*w*/*w*) HAE ointment.

^1^ *p* < 0.001. ^2^ *p* < 0.05.

### 3.6. Antibacterial and Antifungal Activities

The lowest concentration of the extract that prevents the visible growth of each of the test microorganisms was determined as the MIC. As shown in Table 7, the 80% methanol extract of *A. schimperianum* tuber inhibited the growth of all six bacterial strains tested. However, Gram‐positive bacteria appeared to be more susceptible to the extract than the Gram‐negative ones. The hydroalcoholic extract showed an MIC value of 2 mg/mL against MRSA, while the highest MIC value (16 mg/mL) was obtained against *P. mirabilis*. The Gram‐positive bacteria tested were also found to be the most inhibited bacterial pathogens by the solvent fractions. The water fraction showed the strongest activity against MRSA (MIC = 0.5 mg/mL), while *S. epidemidis* was most susceptible to the *n*‐hexane fraction (0.5 mg/mL). However, neither the hydroalcoholic extract nor the solvent fractions of *A. schimperianum* displayed activity against the fungal strains tested at the tested doses.

**Table 7:** Minimum inhibitory concentrations (MICs) of the 80% methanolic tuber extract and solvent fraction of *Arisaema schimperianum*.

**Test substance**	**Growth in nutrient agar containing different concentrations (mg/mL) of the solvent fractions**							
	***Escherichia coli***	***Proteus mirabilis***	***Pseudomonas aeruginosa***	***Staphylococcus aureus***	**MRSA**	***Staphylococcus epidermidis***	***Candida albicans***	***Aspergillus niger***
HAE	8	16	8	2	2	2	NA	NA
HF	8	16	8	8	8	0.5	NA	NA
CF	8	16	8	8	8	2	NA	NA
WF	8	16	4	1	0.5	1	NA	NA
Negative control	NA	NA	NA	NA	NA	NA	NA	NA

*Note:* Negative control: 5% Tween 80; + growth, − no growth; MIC of bacteria positive control (ciprofloxacin: 0.6 *μ*g/mL); MIC of fungi positive control (ketoconazole: 12.5 *μ*g/mL).

Abbreviations: CF, chloroform fraction; HAE, 80% methanolic tuber extract of *Arisaema schimperianum*; HF, *n*‐hexane fraction; MRSA, m*et*hicillin‐resistant *Staphylococcus aureus*; NA, not active; WF, water fraction.

## 4. Discussion

Wound infection remains a major healthcare challenge, particularly in developing countries like Ethiopia, where comorbidities complicate healing [19]. Herbal ointments are considered safe, effective, and affordable for wound treatment [24]. In Ethiopia, the tuber of *A. schimperianum* is traditionally used for treating wounds, glandular tuberculosis, ringworm, scabies, and skin lesions [9, 10]. This study evaluated the wound healing activity of the 80% methanolic extract of *A. schimperianum*. The hydroalcoholic extract was subsequently fractionated with solvents of varying polarity to identify the fraction with the strongest activity and facilitate the isolation of the active compound. Acute oral and dermal toxicity studies showed that the hydroalcoholic extract was nontoxic to mice at an oral dose of 2000 mg/kg. Topical application of 5% and 10% (*w*/*w*) ointments was also safe, with mild irritation observed at the higher concentration, likely due to a stronger dose‐dependent effect [25]. Similar studies have also reported that at a higher concentration, topical application of the tuber extract of *Arisaema leschenaultii* causes skin irritation [26].

For a plant‐based treatment to be scientifically supported as a wound healing agent, it should influence at least two different wound healing processes [27]. Ointments formulated with 5% and 10% (*w*/*w*) of 80% methanolic extract of *A. schimperianum* tuber enhanced wound contraction starting from the 10th and 4th postwounding day, respectively, and increased wound breaking strength, indicating a genuine wound healing effect. In addition, the 10% formulation promoted faster formation of new epithelial tissue and required greater force to break compared to the 5% formulation. This effect is likely due to the higher concentration of bioactive constituents in the 10% ointment, which provided greater local availability of active compounds at the wound site and thereby enhanced tissue repair in a dose‐dependent manner [28, 29].

Further fractionation of the hydroalcoholic extract of *A. schimperianum* tuber revealed that the water fraction was more effective in promoting wound contraction, reducing epithelialization time, and increasing tensile strength compared to the organic solvent fractions. Histological studies confirmed that the water fraction enhanced healing by reducing inflammation, boosting collagen and fibroblast activity, and promoting tissue maturation. These findings align with previous studies, which demonstrated that polar compounds with anti‐inflammatory, antioxidant, and antimicrobial properties support wound healing [30]. Moreover, water‐soluble compounds are generally more easily incorporated into aqueous biological environments, which may aid their availability at the wound site [31]. Hydroxyproline is a unique amino acid predominantly found in collagen. Therefore, it is a common biochemical approach to estimate collagen content by determining hydroxyproline concentration [32]. The ointment prepared from 10% (*w*/*w*) water fraction increased hydroxyproline content more than that of the positive control, suggesting a marked enhancement in collagen synthesis and turnover, which are crucial for wound healing [33].

It is well known that wound infection is detrimental to wound healing and is the most common cause of impairing the wound healing process. In this study, the hydroalcoholic extract and solvent fractions of *A. schimperianum* displayed antibacterial activity, showing a stronger inhibitory effect against Gram‐positive bacteria compared to Gram‐negative bacteria. This is probably due to the difference in cell wall structure between Gram‐positive and Gram‐negative bacteria. Gram‐negative bacteria have a thin peptidoglycan layer and an outer membrane composed of lipopolysaccharides, which can make it more difficult for antimicrobial compounds to penetrate compared to Gram‐positive bacteria [34]. The water fraction of *A. schimperianum* displayed a strong effect against *S. aureus* (MIC = 1 mg/mL), the most prevalent causative organisms associated with infected wounds [35, 36]. Interestingly, the water fraction was most active against MRSA (MIC = 0.5 mg/mL), which is among the most dangerous resistant microorganisms responsible for skin infections [36]. The weaker effect of this polar fraction against the Gram‐negative bacteria implied that the active hydrophilic compound(s) present in it struggled to cross the outer lipophilic membrane. Previous studies carried out on *Arisaema jacquemontii* showed that the methanol extracts of the leaves and tubers possess activity (MIC = 0.37 mg/mL) against *S. aureus* [35]. As plant extracts with MIC values between 100 and 625 *μ*g/mL are regarded as moderately active antibacterial agents [37], the tuber extracts of *A. schimperianum* have the potential to be used as sources of antibacterial compounds. Previous studies by Bhat et al. [38] demonstrated that the methanol extract of *Arisaema utile* tuber inhibits the growth of both bacteria and fungi. However, in the present study, both the hydroalcoholic extract and solvent fractions of *A. schimperianum* tuber failed to show any antifungal activity at the tested doses. Further phytochemical studies are in progress to isolate, characterize, and identify the specific active compounds in this plant responsible for wound healing and antimicrobial activity.

## 5. Conclusion

In conclusion, the present findings disclosed that the tubers of *A. schimperianum* possess wound healing properties, lending scientific support to folkloric or anecdotal use of the plant for the treatment of wounds. The results further confirmed that the aqueous fraction is the most active, suggesting that the active components are polar compounds. It can also be concluded that the antibacterial activity of the plant might be among the contributing factors involved in the enhancement of the wound healing process. In addition, the safety of the hydroalcoholic extract at a high dose and minimal topical irritation in the acute dermal toxicity study ensures its potential as a natural wound therapy.

## Ethics Statement

The animal study protocol was approved by the Institutional Review Board of the School of Pharmacy, College of Health Sciences, Addis Ababa University (Approval Code ERB/SOP/620/16/2024).

## Disclosure

All authors have read and agreed to the published version of the manuscript.

## Conflicts of Interest

The authors declare no conflicts of interest.

## Author Contributions

B.A. and K.A. designed the research studies. B.A. carried out the experiments and drafted the original manuscript. K.A. and D.B. supervised the research work. A.A. and S.D. carried out the antimicrobial tests. B.G. carried out the histopathological analysis. B.A., D.B., and K.A. analyzed the data. D.B. reviewed the statistical analysis. K.A. and D.B. reviewed and edited the manuscript.

## Funding

The research was partially funded by the School of Graduate Studies, Addis Ababa University.

## Data Availability

The data associated with this study are included within the article.

## References

[1] Velnar T. , Baliley T. , and Smrkolj V. , The Wound Healing Process: An Overview of the Cellular and Molecular Mechanisms, Journal of International Medical Research. (2009) 37, no. 5, 1528–1542, 10.1177/147323000903700531, 2-s2.0-70449659580. 19930861

[2] Järbrink K. , Sönnergren H. , Schmidtchen H. , Pang C. , Bajpai R. , and Car J. , Prevalence and Incidence of Chronic Wounds and Related Complications: A Protocol for a Systematic Review, Systematic Reviews. (2016) 5, no. 1, 10.1186/s13643-016-0329-y, 2-s2.0-84986275965. PMC5017042 27609108

[3] Nussbaum S. R. , Carter M. J. , Fife C. E. , DaVanzo J. , Haught R. , Nusgart M. , and Cartwright D. , An Economic Evaluation of the Impact, Cost and Medicare Policy Implications of Chronic Nonhealing Wounds, Value in Health. (2018) 21, no. 1, 27–32, 10.1016/j.jval.2017.07.007, 2-s2.0-85029572227, 29304937. 29304937

[4] Thakur R. , Jain N. , Pathak R. , and Sandhu S. S. , Practices in Wound Healing Studies of Plants, Evidence-Based Complementary and Alternative Medicine. (2011) 2011, 438056, 10.1155/2011/438056, 2-s2.0-80052776616. 21716711 PMC3118986

[5] Gupta A. , Upadhyay N. K. , Sawhney R. C. , and Kumar R. , A Polyherbal Formulation Accelerates Normal and Impaired Diabetic Wound Healing, Wound Repair and Regeneration. (2008) 16, no. 6, 784–790, 10.1111/j.1524-475X.2008.00431.x, 2-s2.0-54949132506, 19128249. 19128249

[6] Khan M. S. A. and Ahmad I. , Khan M. S. A. , Ahmad I. , and Chattopadhyay D. , Chapter 1 - Herbal Medicine: Current Trends and Future Prospects, New Look to Phytomedicine, 2019, Academic Press, 3–13, 10.1016/B978-0-12-814619-4.00001-X.

[7] Mayo S. J. and Gilbert M. G. , A Preliminary Revision of *Arisaema* (Araceae) in Tropical Africa and Arabia, Kew Bulletin. (1986) 41, no. 2, 261–278, 10.2307/4102929.

[8] Hazo H. and Yirgalem A. , Proximate Composition of Qolxo (*Arisaema Schimperianum* Schott) and Different Processing Effect on its Composition, Journal of Food Processing and Technology. (2021) 12, no. 8.

[9] Getachew S. , Medhin G. , Asres A. , Abebe G. , and Ameni G. , Traditional Medicinal Plants Used in the Treatment of Tuberculosis in Ethiopia: A Systematic Review, Heliyon. (2022) 8, no. 5, e09478, 10.1016/j.heliyon.2022.e09478. 35647341 PMC9130528

[10] Yineger H. , Kelbessa E. , Bekele T. , and Lulekal E. , Plants Used in Traditional Management of Human Ailments at Bale Mountains National Park, Southeastern Ethiopia, Journal of Medicinal Plants Research. (2008) 2, no. 6, 132–153.

[11] Dhakal L. , Aryal B. , Joshi G. P. , and Pant D. R. , Nutritional Potential of Selected Species of *Arisaema* Mart. from Nepal, Biodiversitas. (2020) 21, no. 12, 5703–5709.

[12] Hazo H. , Shah G. H. , and Murimi M. , Oxalate and Mineral Composition of Amoche (*Arisaema Schimperianum* S) as Influenced by Different Processing, Journal of Nutrition & Food Sciences. (2021) 11, no. 7.

[13] National Research Council (US) Committee for the Update of the Guide for the Care and Use of Laboratory Animals , Guide for the Care and Use of Laboratory Animals, 2011, 8th edition, National Academies Press. 21595115

[14] British Pharmacopoeia Commission, British Pharmacopoeia, vol. 3, London: The Stationery Office; 2022, pp. 1–1532.

[15] Organisation for Economic Co-operation and Development, OECD Guideline for the Testing of Chemicals; Acute Oral Toxicity up and Down Procedure, Paris, France: OECD Publishing; 2022.

[16] Organisation for Economic Co-operation and Development, OECD Guideline for the Testing of Chemicals: Acute Dermal Toxicity, Paris, France: OECD Publishing; 2017.

[17] Yesuf A. and Asres K. , Wound Healing Activity Guided Isolation of Active Constituents From the Leaves of *Allophylus Abyssinicus* , Phytopharmacology. (2013) 4, no. 2, 442–453.

[18] Nagar H. K. , Srivastava A. K. , Srivastava R. , Kurmi M. L. , Chandel H. S. , and Ranawat M. S. , Pharmacological Investigation of the Wound Healing Activity of *Cestrum nocturnum* (L.) Ointment in Wistar Albino Rats, Journal of Pharmaceutics. (2016) 2016, 9249040, 10.1155/2016/9249040, 27018126. 27018126 PMC4785265

[19] Belachew T. F. , Asrade S. , Geta M. , and Fentahun E. , *In Vivo* Evaluation of Wound Healing and Anti-Inflammatory Activity of 80% Methanol Crude Flower Extract of *Hagenia Abyssinica* (Bruce) J.F. Gmel in Mice, Evidence-Based Complementary and Alternative Medicine. (2020) 2020, 9645792, 10.1155/2020/9645792. 32308725 PMC7149342

[20] Süntar I. P. , Akkol E. K. , Yilmazer D. , Baykal T. , Kirmizibekmez H. , Alper M. , and Yeşilada E. , Investigations on the *in Vivo* Wound Healing Potential of *Hypericum perforatum* L, Journal of Ethnopharmacology. (2010) 127, no. 2, 468–477, 10.1016/j.jep.2009.10.011, 2-s2.0-73749086530, 19833187. 19833187

[21] Clinical and Laboratory Standards Institute, Performance Standards for Antimicrobial Susceptibility Testing: CLSI Supplement M100, 30th ed., vol. 40, no. 1, Wayne, PA: CLSI; 2020.

[22] Alemu M. , Lulekal E. , Asfaw Z. , Warkineh B. , Debella A. , Abebe A. , Degu S. , and Debebe E. , Antibacterial Activity and Phytochemical Screening of Traditional Medicinal Plants Most Preferred for Treating Infectious Diseases in Habru District, North Wollo Zone, Amhara Region, Ethiopia, PLoS One. (2024) 19, no. 3, e0300060, 10.1371/journal.pone.0300060. 38442129 PMC10914283

[23] Tabassum S. , Zia M. , Esperanza J. , Blanco E. J. , Batool R. , Aslam R. , Hussain S. , Wali Q. , and Gulzar M. M. , Phytochemical, *In-Vitro* Biological and Chemo-Preventive Profiling of *Arisaema Jacquemontii* Blume Tuber Extracts, BMC Complementary and Alternative Medicine. (2019) 19, no. 1, 10.1186/s12906-019-2668-4, 2-s2.0-85072227721. PMC6744708 31521162

[24] Pastar I. , Stojadinovic O. , Yin N. C. , Ramirez H. , Nusbaum A. G. , Sawaya A. , Patel S. B. , Khalid L. , Isseroff R. R. , and Tomic-Canic M. , Epithelialization in Wound Healing: A Comprehensive Review, Advances in Wound Care. (2014) 3, no. 7, 445–464, 10.1089/wound.2013.0473. 25032064 PMC4086220

[25] Ali H. and Yaqoob U. , Traditional Uses, Phytochemistry, Pharmacology and Toxicity of *Arisaema* (Araceae): A Review, Bulletin of the National Research Centre. (2021) 45, no. 1, 10.1186/s42269-021-00489-y.

[26] Suruse P. , Kale M. , Gunde M. , Amnerkar N. , and Pathak A. K. , Evaluation of Wound Healing Activity of *Arisaema leschenaultii* Blume in Rats, Der Pharmacia Lettre. (2011) 3, no. 4, 200–206.

[27] Gebrehiwot M. , Asres K. , Bisrat D. , Mazumder A. , Lindemann P. , and Bucar F. , Evaluation of the Wound Healing Property of *Commiphora Guidottii* Chiov. ex. Guid, BMC Complementary and Alternative Medicine. (2015) 15, no. 1, 10.1186/s12906-015-0813-2, 2-s2.0-84939538379. PMC4538748 26283230

[28] Chhabra S. , Chhabra N. , Kaur A. , and Gupta N. , Wound Healing Concepts in Clinical Practice of OMFS, Journal of Maxillofacial and Oral Surgery. (2017) 16, no. 4, 403–423, 10.1007/s12663-016-0880-z, 2-s2.0-85060330890. 29038623 PMC5628060

[29] Deshmukh P. T. , Fernandes J. , Atul A. , and Toppo E. , Wound Healing Activity of *Calotropis gigantea* Root Bark in Rats, Journal of Ethnopharmacology. (2009) 125, no. 1, 178–181, 10.1016/j.jep.2009.06.007, 2-s2.0-67651095600. 19539020

[30] Dev S. K. , Choudhury P. K. , Srivastava R. , and Sharma M. , Antimicrobial, Anti-Inflammatory and Wound Healing Activity of Polyherbal Formulation, Biomedicine and Pharmacotherapy. (2019) 111, 555–567, 10.1016/j.biopha.2018.12.075, 2-s2.0-85059202129, 30597309. 30597309

[31] Fu H. , Wu T. H. , Ma C. P. , and Yen F. L. , Improving Water Solubility and Skin Penetration of Ursolic Acid Through a Nanofiber Process to Achieve Better in Vitro Anti-Breast Cancer Activity, Pharmaceutics. (2024) 16, no. 9, 10.3390/pharmaceutics16091147. PMC11434903 39339184

[32] Chidambara Murthy K. N. , Reddy V. K. , Veigas J. M. , and Murthy U. D. , Study on Wound Healing Activity of *Punica granatum* Peel, Journal of Medicinal Food. (2004) 7, no. 2, 256–259, 10.1089/1096620041224111, 2-s2.0-3042712412. 15298776

[33] Childs D. R. and Murthy A. S. , Overview of Wound Healing and Management, Surgical Clinics of North America. (2017) 97, no. 1, 189–207, 10.1016/j.suc.2016.08.013, 2-s2.0-84998567553. 27894427

[34] Breijyeh Z. , Jubeh B. , and Karaman R. , Resistance of Gram-Negative Bacteria to Current Antibacterial Agents and Approaches to Resolve it, Molecules. (2020) 25, no. 6, 10.3390/molecules25061340. PMC7144564 32187986

[35] Bala K. , Rana J. , and Sagar A. , Antibacterial and Antioxidant Potential of *Arisaema jacquemontii* Blume from Manali, Himachal Pradesh, Bulletin of Pure and Applied Sciences Section-B-Botany. (2019) 38b, no. 1, 23–33, 10.5958/2320-3196.2019.00004.1.

[36] Puca V. , Marulli R. Z. , Grande R. , Vitale I. , Niro A. , Molinaro G. , Prezioso S. , Muraro R. , and Di Giovanni P. , Microbial Species Isolated From Infected Wounds and Antimicrobial Resistance Analysis: Data Emerging From a Three-Years Retrospective Study, Antibiotics. (2021) 10, no. 10, 10.3390/antibiotics10101162. PMC8532735 34680743

[37] Zouine N. , El Ghachtouli N. , El Abed E. , and Koraichi S. I. , A Comprehensive Review on Medicinal Plant Extracts as Antibacterial Agents: Factors, Mechanism Insights and Future Prospects, Scientific African. (2024) 26, e02395, 10.1016/j.sciaf.2024.e02395.

[38] Bhat A. H. , Alia A. , Rather G. M. , and Kumar M. , Isolation and Characterisation of Beta-sitosterol From the Rhizomes of *Arisaema utile* and its Evaluation for Antioxidant Activity, International Journal of Scientific Research in Biological Sciences. (2019) 6, no. 2, 111–118, 10.26438/ijsrbs/v6i2.111118.

